# Response to “Quantifying the health impacts of ambient air pollutants: methodological errors must be avoided”

**DOI:** 10.1007/s00038-016-0808-x

**Published:** 2016-04-26

**Authors:** Marie-Eve Héroux, Bert Brunekreef, H. Ross Anderson, Richard Atkinson, Aaron Cohen, Francesco Forastiere, Fintan Hurley, Klea Katsouyanni, Daniel Krewski, Michal Krzyzanowski, Nino Künzli, Inga Mills, Xavier Querol, Bart Ostro, Heather Walton

**Affiliations:** 1 WHO European Centre for Environment and Health, WHO Regional Office for Europe, Bonn, Germany; 2 Institute for Risk Assessment Sciences, Universiteit Utrecht, Utrecht, The Netherlands; 3 Julius Center for Primary Care and Health Sciences, University Medical Center Utrecht, Utrecht, The Netherlands; 4 MRC-PHE Centre for Environment and Health, London, UK; 5 Health Effects Institute, Boston, USA; 6 Dipartimento di Epidemiologia, ASL Roma E, Rome, Italy; 7 Institute of Occupational Medicine, Riccarton, Edinburgh, UK; 8 Department of Hygiene, Epidemiology and Medical Statistics, University of Athens Medical School, Athens, Greece; 9 McLaughlin Centre for Population Health Risk Assessment, University of Ottawa, Ottawa, ON Canada; 10 Environmental Research Group, King’s College London, London, UK; 11 Swiss Tropical and Public Health Institute, Basel, Switzerland; 12 Public Health England, Centre for Radiation, Chemicals and Environmental Hazards, Chilton, Didcot, Oxfordshire, UK; 13 Institute of Environmental Assessment and Water Research (IDÆA), Consejo Superior de Investigaciones Científicas (CSIC), Barcelona, Spain; 14 Office of Environmental Health Hazard Assessment (OEHHA), Oakland, USA; 15 NIHR Biomedical Research Centre at Guy’s and St. Thomas’ NHS Foundation Trust and King’s College London, London, UK

**Keywords:** Total Mortality, Attributable Fraction, Excess Death, Atomic Bomb Survivor, Excess Case

We thank Morfeld and Erren for their interest in our recent publication on “Quantifying the health impacts of ambient air pollutants: recommendations of a WHO/Europe project” (Héroux et al. 2015). Morfeld and Erren claim that there are potential problems with the statistical approach used in our paper to measure the impact on mortality from air pollution. In fact, they state that “Greenland showed that a calculation based on RR estimates, as performed in the EU research project, does estimate excess cases numbers—but it does not estimate the number of premature cases or etiological cases” (Greenland 1999).

Close reading of the Greenland (1999) paper reveals that he distinguishes three categories of cases occurring in the exposed, observed over a certain period of time: A0, cases which would have occurred anyway even in the absence of exposure—these would typically be estimated from the number of cases occurring in an unexposed control population; A1, cases that would have occurred anyway but were accelerated by exposure; and A2, cases which would not have occurred, ever, without exposure. The word ‘premature’ does not exist in Greenland’s paper, but we consider ‘premature’ and ‘accelerated’ to be the same here. What we usually call the attributable fraction among the exposed is equivalent to the attributable risk (RR−1)/RR which in Greenland’s paper is denoted as the etiologic fraction, (A1 + A2)/(A0 + A1 + A2). And then, etiologic cases are A1 + A2, and excess cases are A2. So, contrary to what Morfield and Erren write, the calculation as performed in our paper estimates etiologic cases (if we follow Greenland’s notation) and not excess cases. After all, in our epidemiology we cannot easily distinguish the excess cases from the accelerated cases.

But let us now take this one step further. Really, the distinction between excess cases and accelerated cases only makes sense for morbidity endpoints or for cause-specific mortality. One can envisage that some of the smokers who developed heart disease over some period of time would have developed it anyway, even in the absence of smoking, after the period of observation. We can only estimate this number A1 when we have observations of heart disease incidence in controls over a more extended period of time. Similarly, some of the smokers dying from heart disease during the period of observation might have died from heart disease anyway, but after a longer period of time. Note that the excess deaths due to heart disease A2, which would never have occurred in the smokers if they had not smoked, necessarily need to be compensated among the controls by an increase in deaths due to some other cause, as in the end, everyone dies. But for total mortality—which is where the bulk of our project’s burden estimates are based on—there are no excess cases (everybody dies in the end); so the estimates based on RR actually correctly estimate the ‘accelerated’ = ‘premature’ cases because the etiologic cases are now equivalent to the accelerated cases, in the absence of excess cases.

Interestingly, this was already described by Greenland in his example of total mortality among the A bomb survivors: “One might object that the extreme structure just described is unrealistic. In reality, however, this extremity is exactly what one should expect if the outcome under study is total mortality in a cohort followed for its entire lifetime, such as the cohort of atomic bomb survivors in Japan. Here, everyone experiences the outcome (death), so there are no “all-or-none” cases, yet everyone may also experience damage and consequent loss of years of life (even if only minor and stress related) owing to the exposure.”

This is exactly the point made by Brunekreef et al. (2007) and we note that this paper was literally and favorably quoted in a paper mentioned in support of the letter (Erren and Morfeld 2011).

The final point to stress here is that the RRs for total mortality and air pollution in our project were all derived from cohort studies in which the denominator for the number of observed cases is not the number of persons exposed or unexposed, but the person years of observation. This is, of course, for the precise reason mentioned by Greenland: if one follows a cohort until extinction, the proportion of deaths is 1 in the exposed and the unexposed alike. The RRs used in our project therefore essentially estimate the ratio of life expectancies in exposed vs. unexposed over the observation period, as the period of observation is censored at time of death and thus shorter among the exposed (who die sooner) than among the unexposed. When applied to a life table, as some of us have shown already many years ago (Brunekreef 1997; Miller and Hurley 2003), one estimates years of life lost, a major component of the Disability-Adjusted Life Years or DALYs which form the core of the GBD analyses which Morfield and Erren also disqualify as an ‘error’. As is well known, the GBD estimates are also expressed as numbers of deaths attributed to certain risk factors, and these are typically denoted as ‘premature’ deaths precisely because there is no such thing as avoidable or excess deaths when it comes to total mortality.

Therefore, in contrast to Morfeld and Erren’s assertion, our project recommendations do properly take into account methodological considerations with respect to quantification of mortality impacts of air pollution.
